# Dissecting empathy: high levels of psychopathic and autistic traits are characterized by difficulties in different social information processing domains

**DOI:** 10.3389/fnhum.2013.00760

**Published:** 2013-11-13

**Authors:** Patricia L. Lockwood, Geoffrey Bird, Madeleine Bridge, Essi Viding

**Affiliations:** ^1^Division of Psychology and Language Sciences, University College LondonLondon, UK; ^2^MRC Social, Genetic and Developmental Psychiatry Centre, Institute of Psychiatry, Kings College LondonLondon, UK; ^3^Institute of Cognitive Neuroscience, University College LondonLondon, UK

**Keywords:** psychopathy, autism spectrum disorder, alexithymia, empathy, affective resonance, cognitive perspective-taking

## Abstract

Individuals with psychopathy or autism spectrum disorder (ASD) can behave in ways that suggest lack of empathy towards others. However, many different cognitive and affective processes may lead to unempathic behavior and the social processing profiles of individuals with high psychopathic vs. ASD traits are likely different. Whilst psychopathy appears characterized by problems with resonating with others’ emotions, ASD appears characterized by problems with cognitive perspective-taking. In addition, alexithymia has previously been associated with both disorders, but the contribution of alexithymia needs further exploration. In a community sample (*N* = 110) we show for the first time that although affective resonance and cognitive perspective-taking are related, high psychopathic traits relate to problems with resonating with others’ emotions, but not cognitive perspective taking. Conversely, high ASD traits relate to problems with cognitive perspective-taking but not resonating with others’ emotions. Alexithymia was associated with problems with affective resonance independently of psychopathic traits, suggesting that different component processes (reduced tendency to feel what others feel and reduced ability to identify and describe feelings) comprise affective resonance. Alexithymia was not associated with the reduced cognitive perspective-taking in high ASD traits. Our data suggest that (1) elevated psychopathic and ASD traits are characterized by difficulties in different social information processing domains and (2) reduced affective resonance in individuals with elevated psychopathic traits and the reduced cognitive perspective taking in individuals with elevated ASD traits are not explained by co-occurring alexithymia. (3) Alexithymia is independently associated with reduced affective resonance. Consequently, our data point to different component processes within the construct of empathy that are suggestive of partially separable cognitive and neural systems.

## INTRODUCTION

Empathy is the capacity to understand or resonate with the affective experiences of others (Singer and Lamm, 2009). Two important processes that contribute to empathy are (i) being aware of, and resonating with, the feelings of another individual such that the awareness of their emotion drives the same state in oneself (henceforth affective resonance) and (ii) identifying and understanding what another individual is thinking/feeling without a necessary affective response (henceforth cognitive perspective-taking). These processes may differentially characterize psychopathy and autism spectrum disorders (ASDs). Although individuals with either disorder can behave in ways that suggest lack of empathy towards others’ (Blair, 2005; Jones et al., 2010) this may be the result of problems in different social information processing domains.

Psychopathy is a disorder characterized by a lack of empathy, shallow affect, and manipulation of others for own gain (Hare, 2003). Difficulties with affective resonance are often apparent. For example, individuals with psychopathy show reduced physiological response to others’ distress (Blair et al., 1997). Adults with psychopathy and children with psychopathic traits display atypical neural responses to others’ pain (Decety et al., 2013; Lockwood et al., 2013; Marsh et al., 2013). In community samples, high levels of psychopathic traits are related to weaker affective responses to fearful faces and happy stories (Seara-Cardoso et al., 2012, 2013). Taken together, these findings indicate clear difficulties in resonating with others’ emotions in both clinical samples with psychopathy and in community individuals with high levels of psychopathic traits. In contrast, one of the defining features of psychopathy is the ability to successfully manipulate others (Hare, 2003). Thus it might be expected that psychopathy would be associated with typical cognitive perspective-taking. Several studies report no cognitive perspective-taking impairments (Blair et al., 1996; Richell et al., 2003; Dolan and Fullam, 2004; Anastassiou-Hadjicharalambous and Warden, 2008) and even superior ability (Hansen et al., 2008) in individuals with psychopathy or high psychopathic traits. However, others have reported problems with tasks related to cognitive perspective-taking in both incarcerated psychopaths (Brook and Kosson, 2013) and healthy samples with high psychopathic traits (Ali and Chamorro-Premuzic, 2010). One possibility for these mixed findings is that different paradigms vary in their level of affective content, with some purported cognitive perspective-taking measures requiring identification of other people’s feelings, rather than just their thoughts. It could be that negative associations between psychopathic traits and cognitive perspective-taking are driven by problems related to basic affective processing, rather than difficulties in cognitive perspective-taking *per se*. In fact, all studies that have reported that psychopathy/psychopathic traits are associated with poorer cognitive perspective-taking have utilized measures with affective content (e.g., Ali and Chamorro-Premuzic, 2010; Brook and Kosson, 2013) and therefore do not necessarily provide evidence for cognitive perspective-taking impairments in psychopathy.

Autism spectrum disorders are characterized by problems with social interaction, communication, and repetitive behaviors. ASD are also associated with atypical empathic processing (e.g., Baron-Cohen and Wheelwright, 2004). Several decades of research indicates that individuals with ASD have difficulties with cognitive perspective-taking (see Hill and Frith, 2003). The findings from studies assessing processes related to affective resonance in ASD are less consistent. There is evidence of absent sensori-motor resonance when viewing others’ pain in individuals with ASD (Minio-Paluello et al., 2009). However, other studies have shown typical sensori-motor resonance when viewing others in pain (Fan et al., 2013) and appropriate physiological responses to others distress (Blair, 1999) in individuals with ASD. When cognitive perspective-taking and empathic concern, a process related to affective resonance, have been compared in individuals with ASD, impairments in cognitive perspective-taking but not empathic concern were found (Dziobek et al., 2008). Some theorists have argued that affective resonance is actually heightened in individuals with ASD (Smith, 2009) and reports of greater empathic facial affect in children with ASD compared to controls supports this (Capps et al., 1993).

A further consideration is the high comorbidity of ASD with alexithymia. Alexithymia is a sub-clinical condition defined by an inability to identify and describe feelings in the self. Preliminary behavioral and neuroimaging research suggests that affective and empathy impairments in ASD may be a function of interoceptive difficulties related to alexithymia rather than ASD *per se* (Silani et al., 2008; Bird et al., 2010) and that after accounting for alexithymia there is no difference in empathy between individuals with ASD and controls (Bird and Cook, 2013). However, one recent fMRI study found no significant moderating effects of alexithymia in an empathy for pain task in individuals with ASD (Fan et al., 2013). Nevertheless, the variance in alexithymia scores was very limited (SD 3.8 in Fan et al., 2013 vs. 11.8 in Bird et al., 2010), which may explain why no effect of alexithymia was observed. Less is known about the possible contribution of alexithymia to empathy impairments seen in individuals with psychopathy. Although the co-occurrence rates of alexithymia and psychopathy are lower than for ASD (Louth et al., 1998), the two disorders do share some common attributes (Lander et al., 2012).

To date, only two studies have directly compared the profile of affective and cognitive processing related to psychopathy and ASD, and these have both been in children. Children with conduct disorder and psychopathic traits showed less affective resonance with others’ emotions but did not have problems with cognitive perspective-taking; conversely, children with ASD showed reduced cognitive perspective-taking but did not have problems with affective resonance (Jones et al., 2010; Schwenck et al., 2012). However, no studies have directly contrasted psychopathic and ASD traits and processes related to affective resonance and cognitive perspective-taking in adults. Moreover, no studies have investigated the contribution of alexithymia to ASD and psychopathic traits in tandem. Psychopathic, ASD and alexithymic traits are present in varying degrees in the general population (Bagby et al., 1994; Baron-Cohen et al., 2001; Hare and Neumann, 2008). Indeed, taxometric studies indicate that psychopathy should be viewed as a dimensional construct that is an extreme variant of normal personality and not a distinct category of behavior (see Hare and Neumann, 2008 for review). Similarly, behavioral genetic studies indicate a similar etiology of autistic traits in the general population as well as in clinical groups (Robinson et al., 2011), thus providing an empirical basis for studying variants in traits associated with these disorders in the general population. Finally, investigating associations between these traits and potential differences in social information processing is one way to dissect the component processes that may contribute to empathy.

Consequently, the present study investigated (i) whether psychopathic and ASD traits were differentially related to performance on affective resonance and cognitive perspective-taking tasks and (ii) whether alexithymia contributes to task performance. We predicted that psychopathic traits would be negatively associated with performance on the affective resonance task but not the cognitive perspective-taking task and that ASD traits would be negatively associated with performance on the cognitive perspective-taking task but not the affective resonance task. Alexithymia has previously been demonstrated to predict empathy deficits while recent neuroimaging results suggest cognitive perspective-taking is unlikely to be affected (Bernhardt et al., 2013). Therefore, we predicted that alexithymia would make a contribution to performance on the affective resonance task, but be unrelated to performance on the cognitive perspective-taking task. We also explored whether the proposed association with alexithymia would reflect variance common to alexithymia and psychopathic traits, or variance unique to alexithymia.

## MATERIALS AND METHODS

### PARTICIPANTS

One hundred and ten healthy adults (50% M; 50% F) aged 18–33 (*M* = 21.9, SD = 3.7) with estimated IQ between 87 and 129 (*M* = 116.8, SD = 8.4) took part. Participants were recruited through university participant databases and the community. All participants provided written informed consent and the study had institutional ethics approval.

### PROCEDURE

Participants completed two tasks to assess affective resonance and cognitive perspective-taking as part of a larger battery of tasks. All tasks were presented in a randomized order followed by the questionnaires.

### EXPERIMENTAL TASKS

#### THEORY OF MIND ANIMATIONS TASK (COGNITIVE PERSPECTIVE-TAKING TASK)

This task assessed participants’ ability to understand others’ complex mental states (e.g., tricking, coaxing) and has been previously used to examine ToM abilities in children with autism (Abell et al., 2000) and healthy participants (Castelli et al., 2002). We selected four “ToM” and four “random” animations from Abell et al. (2000). Each animation featured two characters; a big red and small blue triangle either interacting with one another (ToM animations) or moving randomly (random animations). Participants were asked to watch each animation carefully and to describe what was happening whilst their verbal responses were recorded. Two people transcribed the verbal descriptions that were coded in terms of intentionality and appropriateness. The intentionality scale ranged from 0 (no appreciation of another agent, nor actions or mental states) to 5 (the agent acts with the goal of affecting or manipulating the other agent’s mental states). The appropriateness scale ranged from 0 to 3. One researcher rated all transcriptions and a second researcher rated a random sample of 56. Intra-class correlations (ICC) between raters for intentionality (ICC, single measures = 0.682) and appropriateness (ICC single measures = 0.760) were good. The ratings of intentionality and appropriateness were converted to *z*-scores and a composite score was created.

#### Self-assessment manikin faces task (Affective resonance task)

This task assessed participants’ affective empathic response to emotional faces using the SAM rating scale (Seara-Cardoso et al., 2012). Participants were required to rate their own emotional response to the affective state of another on a nine-point manikin (changing from smiling to a sad face with a neutral expression in the middle) whilst viewing images depicting a person showing either a sad, fearful, angry, happy, or neutral expression. The order of images was randomized for each participant. Ratings for sad, fear, and anger were reverse scored so that the higher scores reflected ratings of greater distress, and thus greater affective resonance, when viewing others’ negative emotions. These variables were then converted to *z*-scores and a composite score was created along with happy ratings.

### QUESTIONNAIRES

#### Self-Report Psychopathy Scale–Short Form (SRP-4-SF, Paulhus et al., in press)

Psychopathic traits were assessed with the SRP-4-SF, a 29-item scale designed to measure psychopathic attributes in non-institutionalized samples. The SRP has been shown to have good construct validity and internal consistency (Cronbach’s alpha 0.89 in the present study) and is strongly correlated with the PCL-R; the clinical measure of psychopathy (Lilienfeld and Fowler, 2006; Paulhus et al., in press). Questions were rated on a five-point scale from “Disagree Strongly” to “Agree Strongly” and included items such as “Most people are wimps” and “I love violent sports and movies.”

#### The Autism Spectrum Quotient (AQ, Baron-Cohen et al., 2001)

Autism spectrum disorder traits were assessed with the AQ, a 50-item scale designed to assess ASD traits in both clinical and community samples. The AQ has good construct validity and internal consistency (Cronbach’s alpha 0.83 in the present study). Questions were rated on a four-point scale from “Definitely Disagree” to “Definitely Agree” and included items such as “I enjoy meeting new people” and “I would rather go to a library than a party.”

#### Toronto Alexithymia scale (TAS, Bagby et al., 1994)

Alexithymic traits were assessed with the TAS, a 20-item scale designed to measure subclinical alexithymic traits. Questions were rated on a five-point scale from “I Strongly Disagree” to “I Strongly Agree” and included items such as “I am often confused about what emotion I am feeling” and “I am often puzzled by sensations in my body.” The TAS has good construct validity and internal consistency (Cronbach’s alpha 0.82 in the present study).

## RESULTS

Performance on the affective resonance and cognitive perspective-taking tasks was positively correlated (*r* = 0.40, *p* < 0.001). All questionnaire measures were also positively correlated with one another (see **Table 1**). First, bivariate correlations were examined to assess whether psychopathic and ASD traits were differentially related to affective resonance and cognitive perspective-taking. As predicted psychopathic traits showed a statistically significant negative correlation with performance on the affective resonance task (*r* = -0.258, *p* = 0.007) whilst ASD traits did not (*r* = -0.102, *p* = 0.291). Conversely, ASD traits showed a statistically significant negative correlation with performance on the cognitive perspective-taking task (*r* = -0.209, *p* = 0.028) whilst psychopathic traits did not (*r* = -0.046, *p* = 0.634).

**Table 1:** Correlations between questionnaire measures of psychopathic, autism spectrum disorder, and alexithymic traits and task performance.

	SRP	AQ	TAS	AR
AQ	0.244*			
TAS	0.252*	0.370**		
AR	-0.258**	-0.102	-0.245*	
CPT	-0.046	-0.209*	-0.120	0.399**

SRP, Self-Report Psychopathy Scale; TAS, Toronto Alexithymia Scale; AQ, Autism Spectrum Quotient; AE, affective resonance task; CPT, cognitive perspective-taking task. *p < 0.05, **p < 0.01.

We conducted hierarchical multiple regression analyses to investigate whether psychopathic and ASD traits were uniquely and differentially related to affective resonance and cognitive perspective-taking, and to examine whether individual differences in alexithymia and/or IQ might explain any associations (see **Table 2**). Two models were run. For the model predicting performance on the affective resonance task, psychopathic traits were entered at the first stage. Psychopathic traits significantly predicted reduced affective resonance (*p* = 0.007). At the second stage ASD traits were entered. Psychopathic traits were uniquely negatively associated with affective resonance (*t* = -2.57, *p* = 0.011) whilst ASD traits were not (*t* = -0.43, *p* = 0.669). The *R*^2^ change was not significant (*F* change = 0.18, *p* = 0.669) indicating that ASD traits did not significantly explain more variance in the model. At the third stage, alexithymia scores were entered. Controlling for alexithymia did not change the pattern of results, but there was a unique negative association between alexithymia and affective resonance (*t* = -1.99, *p* = 0.049), and the *R*^2^ change was significant (*F* = 3.96, *p* = 0.049). At the fourth stage IQ scores were entered. Controlling for IQ did not change the pattern of results, nor was IQ a significant predictor of affective resonance (*p* = 0.73). The same regression sequence was then used for cognitive perspective-taking, but with ASD traits at the first stage and psychopathic traits at the second. ASD traits were significantly negatively associated with cognitive perspective-taking (*t* = -2.22, *p* = 0.028). At the second stage psychopathic traits were entered. ASD traits were uniquely negatively associated with reduced cognitive perspective taking (*t* = -2.16, *p* = 0.033) whilst psychopathic traits were not (*t* = 0.06, *p* = 0.956). The *R*^2^ change was not significant (*F* change = 0.00, *p* = 0.956) indicating that psychopathic traits did not explain significantly more variance in the model. Taking into account alexithymia and IQ did not change the pattern of results, nor did either of these variables predict cognitive perspective-taking. No further *R*^2^ changes were significant (all *F*’s < 1.24, all *p*s > 0.26).

**Table 2:** Hierarchical multiple regression between questionnaire measures of psychopathic, autism spectrum disorder, and alexithymic traits and task performance.

	Affective resonance task				Cognitive perspective-taking task		
	Beta	*t*	*P*		Beta	*t*	*P*
**STEP 1**
SRP	-0.258	-2.772	0.007^*^	AQ	-0.209	-2.224	0.028^*^
**STEP 2**
SRP	-0.248	-2.574	0.011^*^	AQ	-.211	-2.16	0.033^*^
AQ	-0.041	-0.428	0.669	SRP	0.005	0.056	0.956
**STEP 3**
SRP	-0.213	-2.209	0.029^*^	AQ	-0.193	-1.868	0.065^^^
AQ	0.025	0.245	0.807	SRP	0.014	0.144	0.885
TAS	-0.201	-1.991	0.049^*^	TAS	-0.052	-0.501	0.618
**STEP 4**
SRP	-0.218	-2.227	0.028^*^	AQ	-0.196	-1.895	0.061^^^
AQ	0.024	0.236	0.814	SRP	-0.000	0.000	1.000
TAS	-0.200	-1.977	0.051^^^	TAS	-0.050	-0.483	0.630
IQ	0.033	0.353	0.725	IQ	0.106	1.113	0.268

^ *p* < 0.10,

* *p* < 0.05.

*SRP, Self-Report Psychopathy Scale; TAS, Toronto Alexithymia Scale; AQ, Autism Spectrum Quotient; Full IQ calculated from Weschler Intelligence Test of Adult Reading.*

## DISCUSSION

The current study compared associations between psychopathic or ASD traits and tasks assessing affective resonance or cognitive perspective-taking. We demonstrated unique associations between psychopathic traits and reduced affective resonance but not cognitive perspective-taking, and unique associations between ASD traits and reduced cognitive perspective-taking but not affective resonance. Alexithymic traits did not explain observed associations between task performance and psychopathic or ASD traits but rather contributed to performance on the affective resonance task independently of psychopathic traits. This is the first study in healthy adults to show a differential relationship between these variables. Thus, it extends previous findings that have reported contrasting profiles of empathy impairments between children with psychopathic tendencies or ASD (Jones et al., 2010; Schwenck et al., 2012). Our results also suggest that although affective resonance and cognitive perspective-taking measures share some variance, they can capture dissociable processes.

Psychopathy is thought to be characterized by problems with affective resonance but not cognitive perspective-taking. We used measures that were designed to specifically probe affective resonance and cognitive perspective-taking, without there being cognitive perspective-taking demands on the affective resonance task or vice versa. Our results therefore extend and clarify the findings of previous studies reporting reduced affective resonance in individuals high in psychopathic traits (Seara-Cardoso et al., 2012, 2013) by indicating a reduction in affective resonance in the absence of a reduction in cognitive perspective-taking. These data also highlight how high psychopathic traits are not related to atypical cognitive perspective-taking processing when a task without an affective component is used.

Autism spectrum disorders have been consistently linked to problems with cognitive perspective-taking (Hill and Frith, 2003). Interestingly, we found that elevated ASD traits in the general population were also associated with atypical cognitive perspective-taking. In contrast, findings of tasks related to affective resonance processing in autism are mixed, with reduced (Minio-Paluello et al., 2009), intact (Blair, 1999; Dziobek et al., 2008; Bird et al., 2010; Fan et al., 2013), and elevated (Capps et al., 1993) levels of affective processing being reported. Our findings suggest that ASD traits are not associated with either a reduced or an enhanced ability to resonate with the emotions of another, despite the fact that high levels of ASD traits are related to difficulties with understanding others’ minds. It would be useful for future studies to assess multiple forms of processing related to affective resonance, as the paradigms used in some studies that reported intact affective resonance investigated empathic concern, rather than affective resonance. Examining both of these processes in tandem may help to shed further light on the profile of empathic processing in ASD. Moreover, it would also be interesting to further examine the exact cognitive perspective-taking mechanisms that may be disrupted in relation to ASD/high ASD traits. It could be that some disrupted components of cognitive perspective-taking relate to bottom–up processes such as detection of biological movement, whereas others might relate to top–down processes such as the influence of situational cues.

Both psychopathy and ASD have previously been associated with elevated levels of alexithymia (Louth et al., 1998; Lander et al., 2012; Bird and Cook, 2013), and we also observed modest correlations between psychopathic and ASD traits with alexithymia in the present study. Nevertheless, controlling for alexithymic traits did not change the reported associations between psychopathic traits and reduced affective resonance or ASD traits and reduced cognitive perspective-taking. In other words, the reduced ability to identify and describe feelings in the self did not account for the relationship between psychopathic traits and affective resonance or ASD traits and cognitive perspective-taking. The finding that alexithymia did not explain the reduced cognitive perspective-taking abilities characteristic of ASD traits is of particular interest given recent evidence and theory suggesting that alexithymia does account for affective processing deficits related to autism, when they are observed (Bird and Cook, 2013). Our data extend this account by showing that alexithymia does not appear to explain reduced cognitive perspective-taking related to high ASD traits.

We also found that alexithymic traits were negatively associated with a reduction in affective resonance independently of psychopathic traits. This suggests that reductions in affective resonance can be affected both by reduced ability to identify and describe feelings (a characteristic of alexithymia) and a reduced tendency to feel what others feel (a characteristic of psychopathy). The result of independence between psychopathic and alexithymic traits in predicting performance on affective resonance also points to potential component processes within the construct of affective resonance. Future studies could help to determine the mechanisms underlying reduced affective resonance in psychopathy and alexithymia.

A few limitations to the present study should be highlighted. In everyday life empathic responses to others occur in the context of reciprocal social interactions, the present tasks did not present such scenarios in the interest of isolating affective resonance and cognitive perspective-taking demands. Although we chose paradigms to specifically examine two process that contribute to the experience of empathy, these are not exhaustive and further research would benefit from examining a larger collection of tasks that tap a multitude of processes related to empathy. It will also be of interest to determine whether the processing atypicalities associated with psychopathic, ASD, and alexithymia traits explain real life observations of unempathic behavior, as rated by others or observed in an experimental setting. Finally, replication of these results with clinical populations would be informative.

Overall, our findings clarify and extend previous studies examining the profiles of empathy deficits related to psychopathy, ASD, and alexithymia. We show for the first time that in subclinical samples elevated psychopathic traits are related to reduced affective resonance but not cognitive perspective-taking whilst elevated levels of ASD traits are related to reduced cognitive perspective-taking but not affective resonance. Consequently, our data point to different social information processes within the construct of empathy that are suggestive of partially separable cognitive and neural systems.

## Conflict of Interest Statement

The authors declare that the research was conducted in the absence of any commercial or financial relationships that could be construed as a potential conflict of interest.

## AUTHORS CONTRIBUTION

Patricia L. Lockwood, Geoffrey Bird, and Essi Viding designed research, Patricia L. Lockwood and Madeleine Bridge collected data. Patricia L. Lockwood analyzed data, Patricia L. Lockwood, Geoffrey Bird, and Essi Viding wrote paper.
